# Supplementary material for: Asymmetric Synthesis of Quaternary Hydantoins via a Palladium-Catalyzed Aza-Heck Cyclization
Source: J Am Chem Soc. 2025 Nov 14;147(49):44692–8. doi: 10.1021/jacs.5c16022 (PMC12703750; doi:10.1021/jacs.5c16022)
Supplement: Supplementary file 2 [file ja5c16022_si_002.zip › All NMR FID Files/S17/S17_AllNMR/Fluorine/pdata/1/email_TDI01-061 fluorine decoupled_2_1.pdf]

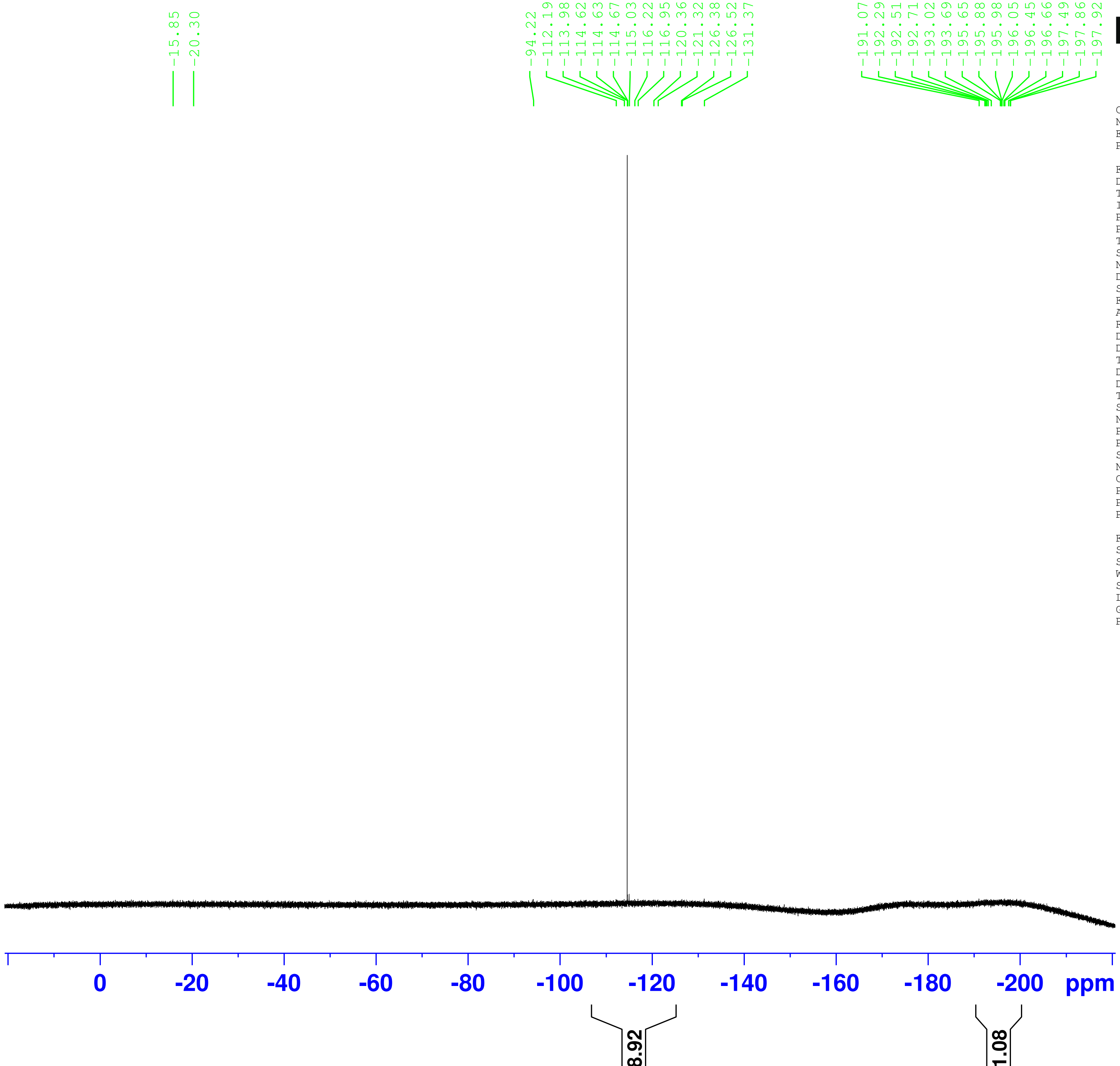


BRUKER

Current Data Parameters

| | |
|---|---|
| NAME | TDI01-061 fluorine decoupled |
| EXPNO | 2 |
| PROCNO | 1 |

F2 - Acquisition Parameters

| | |
|---|---|
| Date_ | 20250219 |
| Time | 21.33 h |
| INSTRUM | Avance Neo 400 U of DE Brown Lab |
| PROBHD | Z104450_0412 ( |
| PULPROG | zgig |
| TD | 131072 |
| SOLVENT | CDCl3 |
| NS | 16 |
| DS | 4 |
| SWH | 90909.091 Hz |
| FIDRES | 1.387163 Hz |
| AQ | 0.7208960 sec |
| RG | 101 |
| DW | 5.500 usec |
| DE | 6.50 usec |
| TE | 298.1 K |
| D1 | 1.00000000 sec |
| D11 | 0.03000000 sec |
| TD0 | 1 |
| SFO1 | 376.4607164 MHz |
| NUC1 | 19F |
| P1 | 15.00 usec |
| PLW1 | 11.81599998 W |
| SFO2 | 400.1316005 MHz |
| NUC2 | 1H |
| CPDPRG[2 | waltz16 |
| PCPD2 | 90.00 usec |
| PLW2 | 8.31639957 W |
| PLW12 | 0.23101000 W |

F2 - Processing parameters

| | |
|---|---|
| SI | 65536 |
| SF | 376.4983662 MHz |
| WDW | EM |
| SSB | 0 |
| LB | 0.30 Hz |
| GB | 0 |
| PC | 1.00 |
